# Rapid erasure of hippocampal memory following inhibition of dentate gyrus granule cells

**DOI:** 10.1038/ncomms10923

**Published:** 2016-03-18

**Authors:** Noelia Madroñal, José M. Delgado-García, Azahara Fernández-Guizán, Jayanta Chatterjee, Maja Köhn, Camilla Mattucci, Apar Jain, Theodoros Tsetsenis, Anna Illarionova, Valery Grinevich, Cornelius T. Gross, Agnès Gruart

**Affiliations:** 1 Mouse Biology Unit, European Molecular Biology Laboratory (EMBL), via Ramarini 32, Monterotondo 00015, Italy; 2 Division of Neuroscience, University Pablo Olavide, Carretera de Utrera—km 1, Sevilla 41013, Spain; 3 Genome Biology Unit, European Molecular Biology Laboratory (EMBL), Meyerhofstraße 1, 69117 Heidelberg, Germany; 4 Schaller Research Group on Neuropeptides, German Cancer Research Center DKFZ, Central Institute of Mental Health, CellNetwork Cluster of Excellence, University of Heidelberg, Im Neuenheimer Feld 581, 69120 Heidelberg, Germany

## Abstract

The hippocampus is critical for the acquisition and retrieval of episodic and contextual memories. Lesions of the dentate gyrus, a principal input of the hippocampus, block memory acquisition, but it remains unclear whether this region also plays a role in memory retrieval. Here we combine cell-type specific neural inhibition with electrophysiological measurements of learning-associated plasticity in behaving mice to demonstrate that dentate gyrus granule cells are not required for memory retrieval, but instead have an unexpected role in memory maintenance. Furthermore, we demonstrate the translational potential of our findings by showing that pharmacological activation of an endogenous inhibitory receptor expressed selectively in dentate gyrus granule cells can induce a rapid loss of hippocampal memory. These findings open a new avenue for the targeted erasure of episodic and contextual memories.

## Abstract (web summary)

Dentate gyrus (DG) is critical for memory formation in the hippocampus but its role in memory retrieval is unclear. Here, Gross and colleagues, show that granule cells in DG are not required for memory retrieval but for maintenance, and inhibiting them with a drug leads to rapid loss of memory.

## Graphical abstract

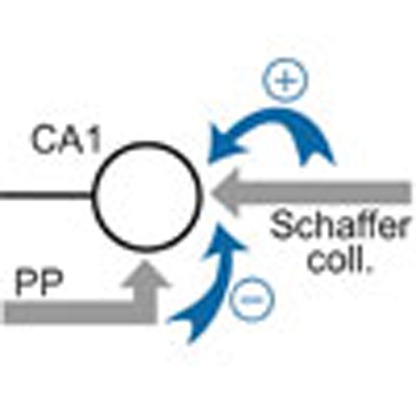

The hippocampus is an evolutionarily ancient part of the cortex that makes reciprocal excitatory connections with neocortical association areas and is critical for the acquisition and retrieval of episodic and contextual memories. The hippocampus has been the subject of extensive investigation over the last 50 years as the site of plasticity thought to be critical for memory encoding. Models of hippocampal function propose that sensory information reaching the hippocampus from the entorhinal cortex via dentate gyrus (DG) granule cells is encoded in CA3 auto-association circuits and can in turn be retrieved via Schaffer collateral (SC) projections linking CA3 and CA1 (refs 1, 2, 3, 4; Fig. 1a). Learning-associated plasticity in CA3–CA3 auto-associative networks encodes the memory trace, and plasticity in SC connections is necessary for the efficient retrieval of this trace^2^,^5^,^6^,^7^,^8^,^9^,^10^. In addition, both CA3 and CA1 regions receive direct, monosynaptic inputs from entorhinal cortex that are thought to convey information about ongoing sensory inputs that could modulate CA3 memory trace acquisition and/or retrieval via SC (refs 11, 12, 13; Fig. 1a). In DG granule cells, sensory information is thought to undergo pattern separation into orthogonal cell ensembles before encoding (or reactivating, in the case of retrieval) memories in CA3 (ref. 14). However, how the hippocampus executes both the acquisition and recall of memories stored in CA3 remains a question of debate with some models attributing a role for DG inputs in memory acquisition, but not retrieval^2^,^15^,^16^,^17^.

Here we combined a pharmacogenetic approach for the rapid and transient suppression of neural activity with *in vivo* electrophysiology during trace eye-blink conditioning to examine the contribution of DG to hippocampal learning and plasticity. Our studies demonstrate that activity in DG granule cells is not necessary for the retrieval of hippocampal memory. Unexpectedly, inhibition of DG is associated with a rapid and persistent loss of memory, as revealed by the suppression of both conditioned responding and learning-associated plasticity. Pharmacogenetic inhibition of entorhinal cortex or local delivery of adenosine A1 receptor antagonist into the hippocampus reverse the suppression of learning-associated plasticity, suggesting a role for direct entorhinal-CA1 inputs in promoting memory loss. Similar memory impairment can be induced by DG inhibition during trace fear conditioning, demonstrating its generalization across hippocampus-dependent memory tasks. Finally, we show that activation of an endogenous inhibitory receptor (neuropeptide Y1 receptor) selectively expressed in DG granule cells can similarly induce rapid and persistent memory loss, opening the possibility of the targeted erasure of hippocampal memories.

## Results

### Rapid pharmacogenetic inhibition of DG granule cells

**Figure 1:**
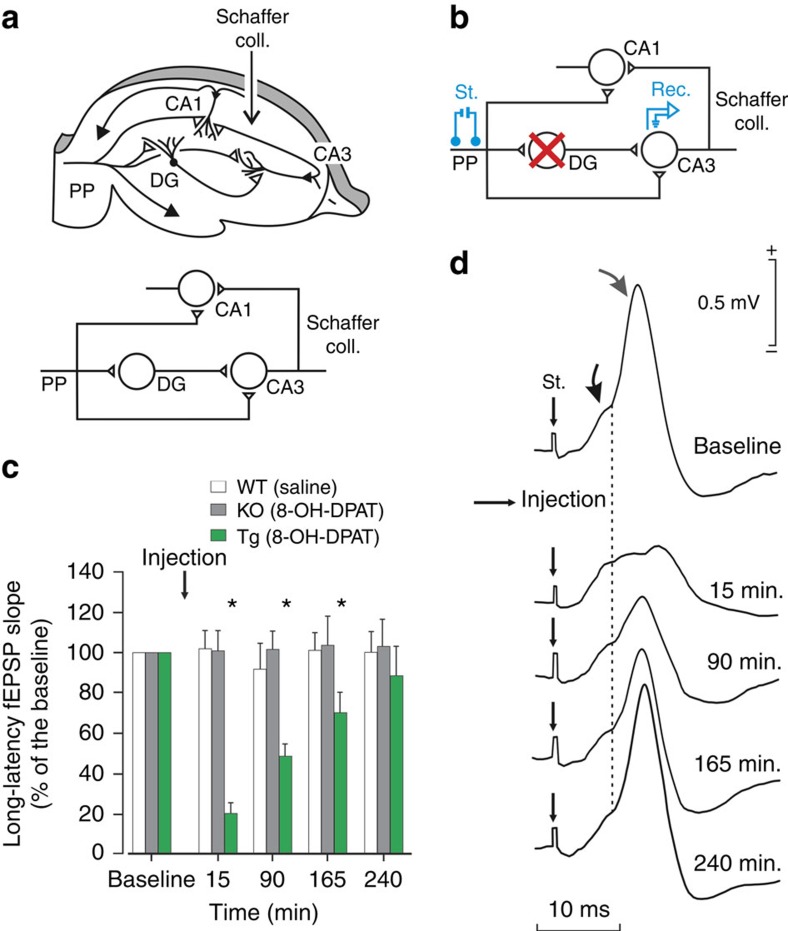
Rapid and selective inhibition of DG neurotransmission *in vivo*. (**a**) The hippocampal tri-synaptic circuit receives PP inputs from entorhinal cortex to DG, CA3 and CA1. (**b**) A stimulating electrode was implanted in the PP and a recording electrode in CA3 pyramidal layer. (**c**) Strength of CA3 pyramidal layer fEPSPs evoked in anaesthetized mice by electrical stimulation of PP inputs showed fast and slow latency population spike components corresponding to direct PP-CA3 and indirect PP–DG-CA3 inputs, respectively. Systemic administration of the selective Htr1a agonist, 8-OH-DPAT (0.3 mg kg^−1^, subcutaneous), to *Htr1a*^DG^ (Tg) mice caused a rapid and selective decrease in the long-latency component that persisted for several hours. Quantification indicated a significant decrease in DG neurotransmission following agonist treatment of *Htr1a*^DG^, but not *Htr1a*^KO^ (KO) littermates or vehicle treated wild-type mice that reached 80% suppression and persisted for >2 h (mean±s.e.m.; *n*=10; *P<0.05; two-way analysis of variance followed by Holm–Sidak *post hoc* test). (**d**) Representative fEPSPs evoked at CA3 pyramidal layer after stimulation of PP inputs before and after agonist treatment. The fast and the slow latency population spike components are indicated (black arrow, short; grey arrow, long).

Until recently, a major limitation in resolving the mechanism of hippocampal function has been the lack of tools that allow for the rapid, transient, efficient and specific inhibition of selected hippocampal cell-types to directly assess their contribution to memory. Here we applied a pharmacogenetic neuronal inhibition strategy that we had previously used to rapidly suppress the firing of DG granule cells in living mice^18^,^19^ to dissect the role of this hippocampal input structure in memory. This pharmacogenetic neural inhibition system depends on the systemic administration of the selective serotonin 1A receptor (Htr1a) agonist, 8-OH-DPAT, to transgenic mice expressing Htr1a exclusively in DG granule cells (*Htr1a*^KO^/*Htr1a*^KO^;*Nrip2*::*Htr1a*/+, called *Htr1a*^DG^). Consistent with previous experiments in brain slices^18^ neurotransmission via DG mossy fibres was rapidly and significantly suppressed in *Htr1a*^DG^ mice treated with 8-OH-DPAT as revealed by an ∼80% reduction of field excitatory postsynaptic potentials (fEPSPs) evoked in CA3 by electrical stimulation of perforant path (PP) inputs (Fig. 1b–d). Importantly, the short latency fEPSP component that reflects direct entorhinal inputs to CA3 was unaffected by 8-OH-DPAT treatment^20^,^21^ (Supplementary Fig. 1; *n*=10, *P*=0.477, two-way analysis of variance followed by Holm–Sidak *post hoc* test). Neither agonist treatment of *Htr1a*^KO^ control littermates nor vehicle treatment of wild-type mice affected synaptic transmission (Fig. 1c). These results demonstrate that the 8-OH-DPAT/Htr1a pharmacogenetic inhibition system is able to rapidly and selectively suppress neurotransmission via DG granule cells *in vivo*.

### DG inhibition induces loss of plasticity and memory

**Figure 2:**
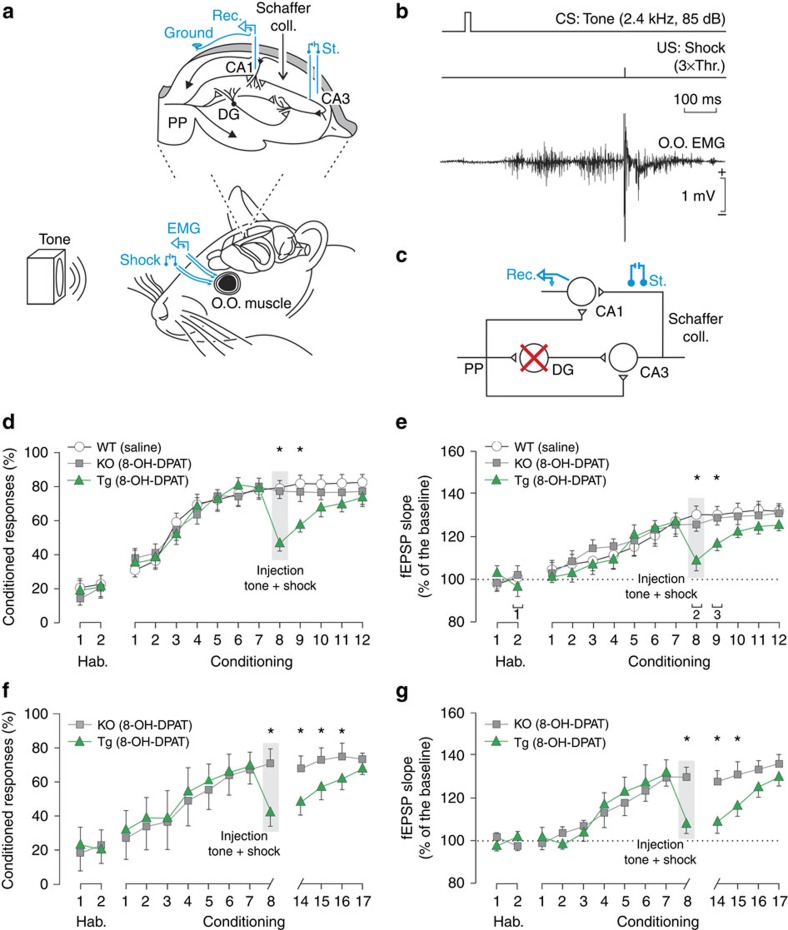
Inhibition of DG induces rapid and persistent loss of hippocampal memory and plasticity. (**a**) Stimulating and recording electrodes were implanted in the SC region and CA1 pyramidal layer, respectively. Eye-blink conditioning was followed using electromyographic (EMG) measurement of orbicularis oculi (O.O.) muscle activity following electrical stimulation of the same muscle. (**b**) EMG was recorded during trace conditioning. *Top:* Conditioned Stimulus (CS), tone, 2.4 kHz, 85 dB. *Middle*: Unconditioned Stimulus (US), shock, 3 × threshold; 500 ms interval. *Bottom*: representative conditioned response in trained animal. (**c**) Evoked fEPSP responses were obtained to stimuli delivered to SC inputs in *Htr1a*^DG^ (Tg) and *Htr1a*^KO^ (KO) control mice. (**d**) Two days of habituation (60 presentations/day, tone only) were followed by 12 days of conditioning (60 presentations/day, 90% tone+shock and 10% tone only) during which animals developed stable conditioned eye-blink responding. A parallel increase in (**e**) SC plasticity was seen during conditioning of wild-type (WT), *Htr1a*^DG^ and *Htr1a*^KO^ mice. Injection of the Htr1a agonist 8-OH-DPAT to *Htr1a*^DG^, but not *Htr1a*^KO^ littermates before conditioning on day 8 lead to a significant reduction in (**d**) conditioned responding and (**e**) SC plasticity. No change in (**d**) conditioned responding or (**e**) plasticity was seen in vehicle treated wild-type mice. Following agonist injection, conditioned behaviour and CA3-CA1 plasticity in the *Htr1a*^DG^ group continued to be significantly reduced and showed a recovery (days 9–12) similar to that seen during initial conditioning (days 1–7). (**f**,**g**) Mice were trained to tone–shock presentations on days 1–8 and 14–17. After treatment with 8-OH-DPAT on day 8, mice were left in their home cages on days 9–13. Both (**f**) conditioned responding and (**g**) SC plasticity remained suppressed in the *Htr1a*^DG^ group when compared with *Htr1a*^KO^ mice on day 14 (mean±s.e.m.; *n*=9–10; *P<0.05; two-way analysis of variance followed by Holm–Sidak *post hoc* test).

To define the contribution of DG to the retrieval of hippocampal memory, we combined our pharmacogenetic strategy with *in vivo* electrophysiology during trace eye-blink conditioning (Fig. 2a–c). We selected trace eye-blink conditioning as a learning paradigm because it is a hippocampus-dependent form of learning in which the neural circuitry supporting conditioned responses is well defined and in which synaptic plasticity correlates are readily identified^5^,^22^. *Htr1a*^DG^ and *Htr1a*^KO^ control littermates that had successfully acquired conditioned behaviour were treated with 8-OH-DPAT (day 8; 0.3 mg kg^−1^, subcutaneous). Agonist-treated *Htr1a*^DG^, but not *Htr1a*^KO^ mice showed a significant decrease in the per cent of successful conditioned responses on the treatment day (Fig. 2d) suggesting a requirement of neural activity in DG granule cells for retrieval of the conditioned response. Unexpectedly, a significant deficit in conditioned responding persisted on the day following agonist treatment in *Htr1a*^DG^ mice (day 9; Fig. 2d) and did not reach control levels before day 12. These data point to a persistent effect of transient DG inhibition on memory recall.

To determine whether the persistent loss of memory on transient DG inhibition was associated with a loss of learning-associated plasticity, we monitored synaptic changes in SC during eye-blink conditioning (Fig. 2a–e and Supplementary Fig. 2). SC synaptic plasticity increased in parallel with eye-blink conditioning and reached an asymptotic mean value of 130% on day 8 of conditioning (Fig. 2e). Surprisingly, 8-OH-DPAT treatment of *Htr1a*^DG^, but not *Htr1a*^KO^ littermates on day 8 was associated with a significant depotentiation of SC (Fig. 2e) demonstrating that maintenance of SC synaptic plasticity was dependent on inputs from upstream DG granule cells. Moreover, SC plasticity continued to be significantly reduced in *Htr1a*^DG^, but not *Htr1a*^KO^ mice on the day following agonist treatment (day 9, Fig. 2e) and did not reach control levels before day 12. Similar results were found in an independent experimental group, demonstrating the robust nature of DG inhibition-induced loss of memory and plasticity (Supplementary Fig. 3). No change in conditioned responding or SC plasticity were observed in *Htr1a*^KO^ mice receiving 8-OH-DPAT or wild-type, *Htr1a*^DG^, or *Htr1a*^KO^ mice receiving saline on day 8 (Supplementary Fig. 3) confirming that the transgene does not interfere with the measured behaviour or plasticity. These observations suggest that the rapid and persistent loss of conditioned responding seen following DG inhibition is caused by a parallel loss of SC plasticity.

Finally, we examined whether the loss of memory and plasticity induced by DG inhibition was persistent. Mice were exposed from day 1 to 8 to tone–shock (conditioned stimulus-unconditioned stimulus, CS-US) pairs and pretreated on day 8 with 8-OH-DPAT to induce DG inhibition. As before, *Htr1a*^DG^, but not *Htr1a*^KO^ mice showed a significant deficit in conditioned responding (Fig. 2f) and SC plasticity (Fig. 2g) on day 8. To test the persistence of these deficits, mice were left in their home cages on days 9–13 and then exposed to paired CS–US presentations from day 14 to 18. Remarkably, both conditioned responding and SC plasticity remained suppressed in *Htr1a*^DG^ mice when compared with *Htr1a*^KO^ littermates on day 14 in a manner indistinguishable from day 8 (Fig. 2f,g). From day 14 to 18 conditioned responding and SC plasticity in *Htr1a*^DG^ mice rose gradually to control levels as a result of learning associated with paired CS–US presentation. These data demonstrate that the rapid loss of memory and plasticity induced by DG inhibition is persistent and that subsequent relearning of the CS–US association follows the same time course as initial learning.

### Memory loss depends on paired CS–US exposure

**Figure 3:**
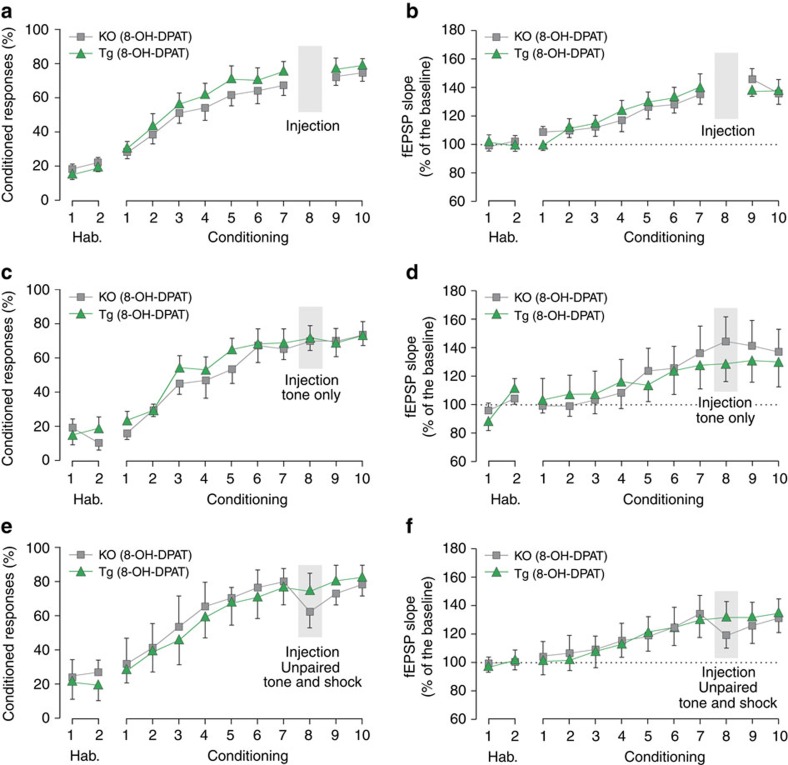
Memory loss depends on paired CS–US presentations. *Htr1a*^DG^ or *Htr1a*^KO^ mice were exposed (**a**,**b**) to tone–shock pairs on days 1–7 and 9–10, but left in the home cage on day 8, (**c**,**d**) to paired tone–shock presentations on days 1–7 and 9–10, but received tone only presentations on day 8, or (**e**,**f**) to tone–shock pairs on days 1–7 and 9–10, but received unpaired tone and shock presentations on day 8. Under these conditions treatment with 8-OH-DPAT on day 8 failed to cause a decrease in (**a**,**c**,**e**) conditioned responding or (**b**,**d**,**f**) fEPSP responses in either *Htr1a*^DG^ or *Htr1a*^KO^ mice (mean±s.e.m.; *n*=9–10; two-way analysis of variance followed by Holm–Sidak *post hoc* test).

To determine whether DG inhibition was sufficient to induce loss of plasticity and memory, we tested mice exposed from day 1 to 7 to tone–shock pairs, but left in the home cage on day 8. Under these conditions DG inhibition on day 8 was not associated with a significant alteration in conditioned responding or SC plasticity (Fig. 3a,b) demonstrating that DG inhibition by itself does not induce synaptic depotentiation or memory loss and is not associated with a general deficit in performance or SC plasticity. Next, we tested whether DG inhibition coupled to tone only exposure on day 8 would induce memory loss. Under these conditions DG inhibition was not associated with a significant alteration in conditioned responding or SC plasticity (Fig. 3c,d). This finding suggests that presentation of both CS and US are necessary during DG inhibition to induce synaptic depotentiation and memory loss. It also shows that DG inhibition does not induce memory loss by enhancing extinction, a form of memory loss and synaptic depotentiation induced by exposure to tone presentation^5^ (Supplementary Fig. 3).

Finally, we examined whether associative pairing of CS and US during DG inhibition was necessary for memory loss. DG inhibition on day 8 was coupled with exposure to explicitly unpaired CS and US presentations, a pseudoconditioning protocol that we have previously shown does not support associative learning^5^. Under these conditions DG inhibition was not able to induce loss of memory or plasticity (Fig. 3e,f) demonstrating a requirement for the associative, paired presentation of CS and US in the memory loss mechanism. Importantly, spectral power of CA1 recordings were unaffected by DG inhibition (Supplementary Fig. 4) ruling out an indirect effect of altered rhythmic activity on SC plasticity. Together, these findings argue that blockade of neural activity in DG unmasks a depotentiation mechanism that rapidly reverses SC plasticity during associative presentation of tone and shock and that causes a persistent impairment in hippocampal memory retrieval.

### Loss of plasticity depends on entorhinal cortex inputs

**Figure 4:**
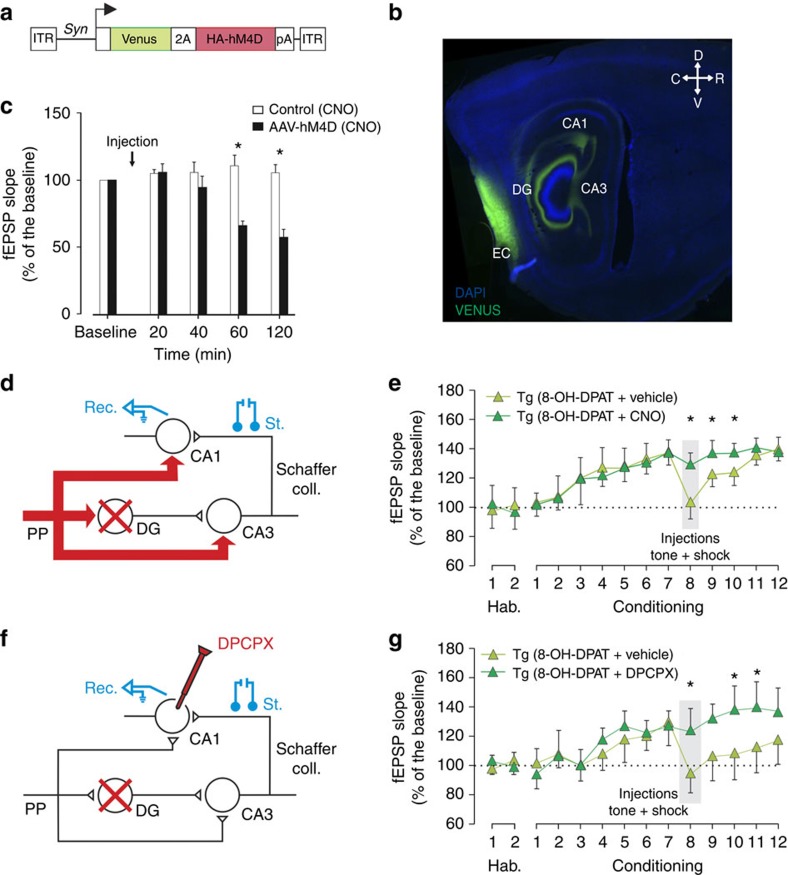
Loss of plasticity depends on entorhinal cortex inputs and local adenosine signalling. (**a**–**c**) Mice locally infected with an (**a**) AAV expressing the Venus fluorescent protein and HA-tagged hM4D DREADD receptor (HA-hM4D) for pharmacogenetic neural inhibition under control of the *Synapsin (Syn)* gene promoter showed expression in (**b**) entorhinal cortex (EC) and PP projections to hippocampus (DG, CA3 and CA1). (**c**) Systemic administration of the selective hM4D agonist CNO (3 mg kg^−1^, intraperitoneal) to wild-type mice caused a significant decrease in fEPSP recorded in CA1 following stimulation of olfactory bulb-entorhinal cortex projections in animals infected with AAV-hM4D, but not in non-infected (control) animals. Significant suppression of fEPSPs was observed 1 h after agonist treatment and persisted for at least 2 h (mean±s.e.m.; *n*=4–7; *P<0.05; two-way analysis of variance followed by Holm–Sidak *post hoc* test). (**d**,**e**) *Htr1a*^DG^ (Tg) mice (**d**) expressing hM4D in the entorhinal cortex and its projections to hippocampus (red arrows) and implanted with stimulating and recording electrodes in the SC and CA1 regions, respectively, were (**e**) subjected to trace eye-blink conditioning with tone–shock presentations on days 1–12. Systemic pre-treatment with the hM4D agonist CNO (3 mg kg^−1^, intraperitoneal) on day 8 reversed the loss of SC plasticity induced by 8-OH-DPAT treatment on that day (mean±s.e.m.; *n*=12; *P<0.05; two-way analysis of variance followed by Holm–Sidak *post hoc* test). (**f**,**g**) *Htr1a*^DG^ mice (**f**) implanted with stimulating and recording electrodes in the SC and CA1 regions, respectively, and bilateral cannulae aimed at dorsal CA1 were (**g**) subjected to trace eye-blink conditioning as above. Pre-treatment on day 8 with the adenosine A1 receptor antagonist, DPCPX (82 nmol) caused a significant reversal of the SC depotentiation induced by 8-OH-DPAT treatment on that day when compared with vehicle pretreated mice. Notably, reversal of DG inhibition-induced plasticity by DPCPX persisted through day 12 (mean±s.e.m.; *n*=7; *P<0.05; two-way analysis of variance followed by Holm–Sidak *post hoc* test).

*In vitro* electrophysiological experiments with hippocampal slices have shown that stimulation of PP inputs to CA1 is able to reverse long-term potentiation induced at SC synapses^11^,^12^,^13^,^23^ suggesting that learning-associated activity in PP inputs to CA1 might be involved in the DG inhibition-induced SC depotentiation. To test this hypothesis we repeated our trace eye-blink conditioning experiments in mice in which activity in entorhinal cortex neurons could be selectively inhibited. *Htr1a*^DG^ mice were bilaterally infected in the entorhinal cortex with adeno-associated virus (AAV) expressing Venus fluorescent protein and the inhibitory hM4D DREADD receptor^24^ under a neuron-specific promoter^25^ (AAV-hSyn::Venus-2A-hM4D; Fig. 4a). Selective expression of Venus reporter in entorhinal cortex neurons and their efferents to DG, CA3 and CA1 was confirmed by immunofluorescence (Fig. 4b). Control electrophysiology experiments in anaesthetized wild-type mice similarly infected with AAV-hSyn::Venus-2A-hM4D and systemically injected with the selective hM4D agonist clozapine-*N*-oxide (CNO (ref. 24)) demonstrated a significant suppression of PP-CA1 neurotransmission, as revealed by a ∼50% reduction of fEPSPs evoked in CA1 by electrical stimulation of the olfactory bulb-entorhinal cortex pathway^26^ (Fig. 4c and Supplementary Fig. 5), although we cannot exclude a contribution of volume conduction from PP–DG inputs to the measured evoked responses. SC synaptic plasticity was monitored in infected *Htr1a*^DG^ mice during trace eye-blink conditioning (Fig. 4d). On day 8 of training, mice were pretreated with either vehicle or CNO to suppress PP-CA1 inputs and administered 8-OH-DPAT to induce SC depotentiation (Fig. 4d). As expected, learning-induced SC plasticity was strongly depotentiated in vehicle-pretreated animals receiving 8-OH-DPAT (Fig. 4e). However, depotentiation was significantly attenuated in CNO-pretreated mice (Fig. 4e). These data support a role for entorhinal cortex inputs to the hippocampus in promoting the depotentiation of SC synaptic plasticity induced by DG inhibition.

### Loss of plasticity involves local adenosine signalling

Hippocampal slice experiments demonstrated that PP stimulation-induced SC depotentiation could be blocked by treatment with an antagonist of adenosine A1 receptors, suggesting that local release of adenosine in response to PP-CA1 stimulation was necessary to induce long-term depression at SC synapses^22^,^27^. To test whether a similar mechanism might underlie the depotentiation of SC synapses seen during DG inhibition *in vivo*, we repeated our earlier experiment with local, bilateral administration of the adenosine A1 receptor antagonist 1,3-dipropyl-8-cyclopentylxanthine (DPCPX) into dorsal CA1 on day 8 (Fig. 4f,g and Supplementary Fig. 6). As expected, *Htr1a*^DG^ mice treated with vehicle showed a marked depotentiation of SC plasticity following DG inhibition on day 8 and this effect was significantly attenuated in mice pretreated with DPCPX (Fig. 4g). Importantly, DPCPX treatment on day 8 was associated with a long-term blockade of SC plasticity deficits on the following days (Fig. 4g). The administration of DPCPX in *Htr1a*^KO^ mice either alone or together with 8-OH-DPAT had no significant effect on plasticity, although there was a trend for DPCPX to increase SC plasticity on the treatment day (Supplementary Fig. 6) suggesting a role for adenosine-dependent depotentiation during undisturbed learning. These data demonstrate that the persistent depotentiation of SC synapses induced by DG inhibition is the result of an adenosine A1 receptor-dependent, long-term depression-like phenomenon in region CA1 and further implicate direct entorhinal inputs to CA1 in SC depotentiation.

### Generalization of memory loss phenomenon

**Figure 5:**
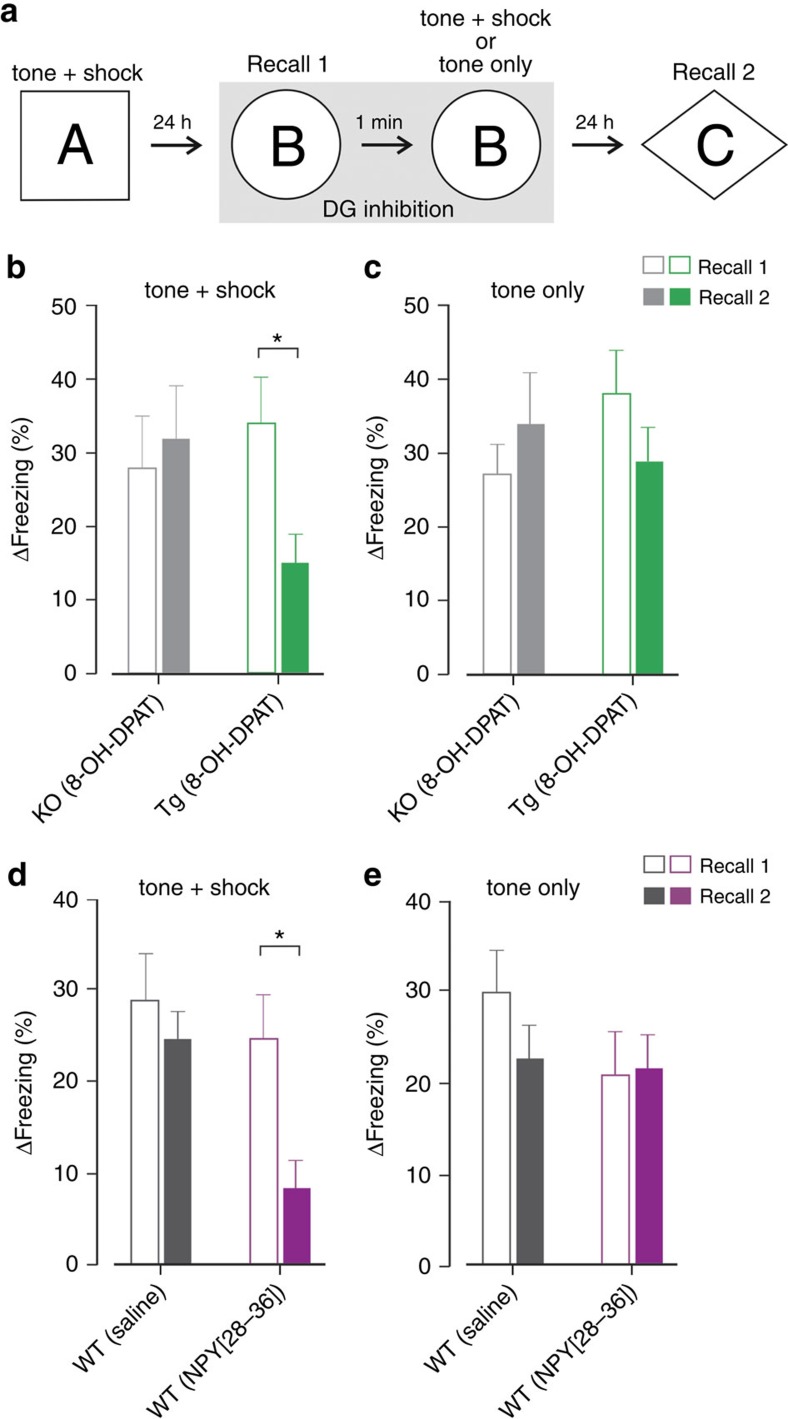
Inhibition of DG induces persistent memory loss during trace fear conditioning. (**a**) Mice received six tone–shock pairings (tone: 20 s, 85 dB, 3 kHz; shock: 2 s, 0.4 mA; 20 s apart) in context A. Twenty-four hours later mice were placed into context B and presented with a tone (60 s) to assess recall (recall 1) after which they were briefly removed, the grid floor covering was taken out, and they were replaced into context B and subjected again to six tone–shock pairings (tone+shock) or six tones only (tone only). Twenty-four hours later mice were placed in context C and presented with a tone (60 s) to assess recall (recall 2). (**b**,**c**) Pre-treatment (0.3 mg kg^−1^, subcutaneous) with 8-OH-DPAT on the second day significantly reduced freezing in *Htr1a*^DG^ (Tg), but not *Htr1a*^KO^ (KO) control mice during recall 2 compared with recall 1 in the (**b**) tone–shock, but not (**c**) tone only conditions (*t*-test; *n*=9–10). (**d**,**e**) Pre-treatment (20 nmoles per mouse, i.c.v.) of wild-type mice on the second day of trace fear conditioning (**a**) with [Pro^30^,Nle^31^,Bpa^32^,Leu^34^]NPY(28–36), but not vehicle significantly reduced freezing during recall 2 compared with recall 1 in the (**d**) tone–shock, but not (**e**) tone only conditions (mean±s.e.m.; *n*=8–14; *P<0.05; *t*-test).

To examine whether DG inhibition-induced memory loss is a general feature of hippocampal learning, we examined the role of DG granule cells in trace fear conditioning, a widely used hippocampus-dependent memory task. *Htr1a*^DG^ and control *Htr1a*^KO^ littermate mice were subjected to trace fear conditioning by placing them into a chamber (context A) and exposing them to a series of paired tone and foot shock stimuli separated by 20 s (Fig. 5a; six tone–shock pairs; tone: 20 s, 3 kHz; shock: 2 s, 0.4 mA). One day later the mice were placed into a novel context (context B) and freezing behaviour during the presentation of the tone was assessed as a measure of fear conditioning recall (recall 1; Fig. 5a). Immediately thereafter, mice were removed from context B and placed back into the same context with an exposed grid floor and subjected to either a second training session (six tone–shock pairs; tone: 20 s, 3 kHz; shock: 2 s, 0.4 mA) or to a series of identical tones without shock (tone only). Mice were pretreated on the second day with 8-OH-DPAT to induce DG inhibition during both recall and subsequent learning. Finally, 1 day later, mice were placed into a third context (context C) to test their recall in the absence of DG inhibition (recall 2; Fig. 5a). Although treatment of *Htr1a*^DG^ mice with 8-OH-DPAT did not affect trace fear conditioning recall (recall 1), it was associated with a significant impairment of recall on the day following treatment (recall 2, Fig. 5b). Importantly, similar treatment of *Htr1a*^DG^ mice exposed only to tones on the second day (Fig. 5c) was not associated with deficient recall, consistent with tone–shock association being required for memory loss. *Htr1a*^KO^ littermates treated with 8-OH-DPAT did not show impaired recall on either day (Fig. 5b,c) confirming a requirement for DG inhibition in the fear conditioning deficit.

### Memory loss can be induced by Npy1r activation

Finally, we asked whether we could induce memory loss by manipulating neural activity in DG granule cells without the need for transgenic pharmacogenetic inhibition technology. Such an approach might offer a promising method for the targeted reversal of unwanted memories in other species, including humans. A search of the Allen Brain Atlas gene expression database^28^ identified a somato-dendritic inhibitory G-protein coupled receptor, Npy1r, expressed selectively in DG granule cells. Although effective small molecule agonists are not presently available for this receptor, at least one peptidic agonist, [Pro^30^,Nle^31^,Bpa^32^, Leu^34^]NPY(28–36), has been reported to selectively activate Npy1r (ref. 29). Control experiments in anaesthetized wild-type mice showed that administration of [Pro^30^,Nle^31^,Bpa^32^, Leu^34^]NPY(28–36), but not vehicle into the cerebral ventricles rapidly and significantly suppressed neurotransmission via DG mossy fibres *in vivo* as revealed by a ∼60% reduction of fEPSPs evoked in CA3 by electrical stimulation of PP inputs (Supplementary Fig. 7). These results show that endogenous Npy1r expressed on DG granule cells can be targeted by systemic agonists to induce DG inhibition in wild-type animals. Next, we repeated our trace fear conditioning experiments to determine whether the Npy1r agonist could induce memory loss. Similar to data obtained with transgenic mice treated with 8-OH-DPAT, wild-type mice receiving the Npy1r agonist, but not those receiving vehicle showed a significant decrease in conditioned freezing on the second recall session (recall 2; Fig. 5d). No deficit in conditioned responding was observed on the first recall day (recall 1) during drug treatment, nor on either day in mice exposed to only tones on the first recall day (tone only; Fig. 5e). These data show that activation of an endogenous inhibitory receptor expressed selectively in DG granule cells can be used to induce learning-associated memory loss, and suggest an avenue for the translation of these findings to other organisms.

## Discussion

In the present study we examined the contribution of DG granule cells to learning and recall and its associated synaptic plasticity in animals that had previously acquired a hippocampal memory. We found that transient pharmacogenetic inhibition of DG granule cells did not impair conditioned responding to CS presentation nor alter SC synaptic plasticity demonstrating that DG is not required for memory recall (Fig. 3c,d). However, when DG inhibition occurred during paired presentation of CS and US, we observed a rapid loss of SC synaptic plasticity and conditioned responding to CS (Fig. 2d,e and Supplementary Fig. 3). Strikingly, the synaptic plasticity and behavioural impairment persisted in the absence of further stimulus presentation and later relearning occurred at a rate indistinguishable from initial learning, suggesting a loss of the memory trace (Fig. 2f,g).

One possible explanation for the memory loss seen on DG inhibition is that presentation of paired CS–US has a dual effect on CA1 plasticity, on the one hand strengthening SC synapses via a DG-dependent mechanism (indirect inputs to CA1 via the tri-synaptic circuit) and on the other hand weakening SC synapses in a non-DG-dependent manner (direct PP-CA1 inputs). This explanation is consistent with several studies in the literature reporting mechanistic and functional differences between the direct and the indirect inputs to CA1 (refs 12, 13, 30, 31, 32). Furthermore, earlier *in vitro*^12^,^23^ and *in vivo*^33^ electrophysiology studies found that stimulation of PP-CA1 inputs to the hippocampus could depotentiate synaptic plasticity that had been previously acquired at SC synapses suggesting that the direct PP pathway might promote depotentiation during hippocampal learning. To test this possibility, we used dual, orthogonal pharmacogenetic inhibition of DG and entorhinal cortex to show that the memory loss phenomenon we observed depended on PP inputs (Fig. 4e). Furthermore, one of the earlier studies^23^ had shown that PP stimulation-induced SC depotentiation could be inhibited by blockade of adenosine A1 receptors, but not several other receptors, and we found that bilateral administration of DPCPX to the CA1 region of the hippocampus blocked synaptic depotentiation in our model (Fig. 4g).

**Figure 6:**
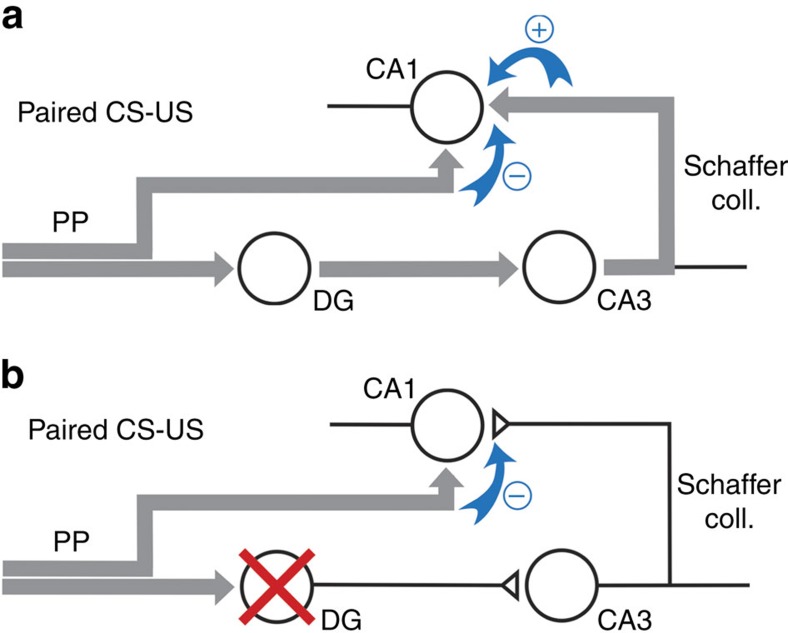
Model for function of PP-CA1 inputs to the hippocampus. Area CA1 of the hippocampus receives information directly from the entorhinal cortex (direct PP-CA1 pathway) and also indirectly via the tri-synaptic circuit. (**a**) Presentation of paired CS–US promotes potentiation of SC synapses (+) via the indirect pathway depotentiation of SC synapses (–) via the PP-CA1 pathway. In an animal having successfully undergone learning, potentiation and depotentiation are balanced, SC synaptic strength is stable and memories can be retrieved. (**b**) Inhibition of DG during CS–US presentation suppresses potentiation via the indirect pathway, unmasking depotentiation of SC synapses and promoting memory loss.

Our data lead us to propose a novel function for PP-CA1 inputs to the hippocampus. During CS–US presentation, but not during presentation of unpaired CS–US or CS alone, information arriving via this pathway actively promotes depotentiation of SC synapses, while information arriving via the DG pathway opposes this depotentiation. Thus, in an animal that has successfully acquired a hippocampal-dependent memory, and in which the direct and indirect pathways are intact, SC synaptic strength is stable and memories can be retrieved. However, when the DG pathway is blocked, as we have done artificially in our study, depotentiation is favoured and memory is lost (see scheme, Fig. 6). The precise function of PP-dependent SC depotentiation remains unclear at this point, but we speculate that it may play a role in weakening previously acquired associations to facilitate the encoding of new memories. Existing data show that selective blockade of synaptic activity in entorhinal cortex neurons projecting to CA1 impairs the acquisition of trace fear conditioning^34^ and support our hypothesis of a positive role for this pathway in learning^13^,^30^,^32^,^33^. Moreover, our DPCPX experiments suggest that blockade of the depotentiation mechanism promotes SC synaptic plasticity during CS–US presentation in otherwise intact animals (Fig. 4g). However, further loss and gain-of-function manipulations of this pathway coupled with *in vivo* electrophysiology and learning behaviour are needed to directly test a role of PP-CA1 inputs in memory clearing.

Our finding that DG granule cells are not required for retrieval of hippocampal memory is consistent with previous data arguing that retrieval of associative information encoded in CA3–CA3 and SC plasticity is achieved via direct PP projections to CA3 (refs 1, 2, 3, 4, 35, 36, 37, 38). However, our data appear to contradict at least one recent study demonstrating a role for DG granule cells in retrieval during contextual fear conditioning^39^. We believe this discrepancy is due to a requirement for DG granule cells in the processing of the contextual CS (ref. 40). However, to rule out the possibility that other methodological differences between the studies underlie the discrepancy, it would be important to determine whether the cell-type specific optogenetic inhibition method used in their study left intact the recall of hippocampal-dependent memories for discrete cues.

Our study raises several questions. First, while we show SC depotentiation is adenosine receptor dependent, the location of adenosine signalling is not clear. Adenosine A1 receptors are expressed highly in CA3 pyramidal cells as well as more modestly in CA1 (ref. 28), and a study in which this receptor was selectively knocked out in one or the other of these structures demonstrated a role for presynaptic CA3, but not postsynaptic CA1 receptors in dampening SC neurotransmission^41^ suggesting a presynaptic mechanism for our effect. The source of adenosine, on the other hand, could involve pre- and/or postsynaptic release as well as release from non-neuronal cells such as astrocytes^27^,^42^. Second, although our DPCPX experiment pointed to a role for PP-CA1 projections in SC depotentiation, our entorhinal cortex pharmacogenetic inhibition experiment did not allow us to distinguish between contributions of PP-CA1 and PP-CA3 inputs. Although we cannot rule out a contribution of PP-CA3 projections to SC depotentiation, earlier *in vitro* and *in vivo* electrophysiology studies clearly demonstrate a role for PP-CA1 in SC depotentiation^12^,^22^,^33^. Third, the method we used to assess SC postsynaptic strength, namely electrical stimulation evoked field potentials does not allow us to rule out that changes in synaptic plasticity at non-SC inputs underlie our plasticity effects. Experiments using targeted optogenetic stimulation of CA3 efferents could be used to more selectively measure SC synaptic strength. Fourth, our observation that SC depotentiation and memory loss occurred only during paired, but not unpaired CS–US presentation (Fig. 2d,e) suggests that the memory loss phenomenon we describe is distinct from other well-described avenues for memory degradation, including enhancement of extinction^43^ and blockade of reconsolidation^44^. Finally, our findings demonstrating generalization of DG inhibition-induced memory loss across tasks coupled with our identification of an endogenous pharmacological target that can induce similar memory loss raise the possibility that the novel memory mechanism we have uncovered may be useful for erasing unwanted memories in a clinical setting.

## Methods

### Animals

Transgenic mice (genetic background: B6J;CBA;129S6/SvEvTac) were littermates derived from breeding *Htr1a*^KO^/*Htr1a*^KO^;*Nrip2*-*Htr1a*/+ and *Htr1a*^KO^/*Htr1a*^KO^;*+*/+ mice^18^ (called *Htr1a*^DG^ and *Htr1a*^KO^, respectively) following protocols approved by the Italian Ministry of Health and shipped to Pablo Olavide University for experimental testing following experimental protocols approved by the University Ethics Committee and the Spanish Ministry of Health and in accordance with the guidelines of the European Union (2010/276:33–79/EU) and Spanish regulations (BOE 34:11370-421, 2013). Wild-type animals used for trace fear conditioning (genetic background: C57BL/6J) were derived from an internal EMBL breeding colony and tested following an experimental protocol approved by the Italian Ministry of Health. Experiments were carried out with male and female littermates that were 3–5 months old. Animals were group housed, switched to individual cages following surgery, and kept on a 12 h light/dark cycle (lights on at 8:00) with constant ambient temperature (21.5±1 °C) and humidity (55±8%). Food and water were available *ad libitum*. All mice that completed experimental protocols in the absence of deteriorated fEPSPs, electroencephalogram and/or electromyographic recordings were included. Additional animals were used in preliminary studies to select the appropriate drug doses and test the stability of recording and stimulating systems.

### Surgery

Mice were anaesthetized with 0.8–1.5% isoflurane, supplied from a calibrated Fluotec 5 vaporizer (Fluotec-Ohmeda, Tewksbury, MA) at a flow rate of 1–2 l min^−1^ oxygen (AstraZeneca, Madrid, Spain) and delivered by a mouse anaesthesia mask (David Kopf Instruments, Tujunga, CA). For trace eye-blink conditioning animals were implanted with bipolar recording electrodes in the left orbicularis oculi muscle and stimulating electrodes on the ipsilateral supraorbital nerve (Fig. 2a). These electrodes were made from 50 μm, Teflon-coated, annealed stainless steel wire (A-M Systems, Carlsborg, WA). For measurement of SC synaptic efficacy animals were implanted with stimulating electrodes in the right SC/commissural pathway of the dorsal hippocampus^45^ (1.5 mm posterior to bregma, 2 mm lateral, and 1–1.5 mm from the brain surface) and with a recording electrode in the right CA1 stratum radiatum (2.2 mm posterior to bregma, 1.2 mm lateral and 1–1.5 mm from the brain surface; Figs 2a,c and 4d,f and Supplementary Fig. 3a). Hippocampal electrodes were made from 50 μm, Teflon-coated, tungsten wire (Advent Research, Eynsham, UK). All the implanted wires were soldered to two four-pin sockets (RS Amidata, Madrid, Spain) and fixed to the skull with dental cement^5^. Recording electrodes were aimed at the apical dendrites of pyramidal CA1 cells^5^,^46^ and their final location was adjusted by the profile of fEPSPs evoked by SC stimulation during surgery. For experiments to determine the activity of DG granule cells, animals were implanted with stimulating electrodes in the perforant pathway (2.2 mm posterior to bregma, 2 mm lateral and 1 mm from the brain surface) and recording electrodes in the pyramidal CA3 area (1.5 mm posterior to bregma, 1.7 mm lateral and 1.3 mm from the brain surface; Fig. 1b). For experiments to inhibit entorhinal-hippocampal neurons, four bilateral injections of AAV aimed at the entorhinal cortex (posterior: −4.8 mm, lateral: ±3 mm to bregma, depth: −3.25 mm and −2.75 mm from Bregma; posterior: −4.6 mm, lateral: ±2.88 mm to bregma, depth: −3.25 mm and −2.75 mm from Bregma; Fig. 4b,d) were performed using a glass pipette (intraMARK, 10–20 μm tip diameter, Blaubrand, Wertheim, Germany) connected to a syringe and a stereotaxic micromanipulator (0.4 μl of AAV-containing solution per hemisphere). Behavioural experiments were performed 2–3 weeks after surgery. To determine the extent of inhibition of entorhinal cortex-CA1 synaptic transmission in AAV infected mice, stimulating electrodes in the olfactory bulb (4.0 mm anterior to bregma, 1.0 mm lateral and 2.2 mm from the brain surface) and recording electrodes in CA1 (2.2 posterior to bregma, 1.2 mm lateral and 1.3 from the brain surface; Fig. 4c) were implanted in a second surgical procedure. For local drug administration animals were bilaterally implanted with a 26-gauge stainless steel guide cannula in dorsal CA1 (Plastics One; 1.94 mm posterior to Bregma, 1.6 mm lateral and 0.7 mm from the brain surface, that is, 0.5 mm above the infusion target; Fig. 4f) or unilaterally implanted in the right lateral ventricle (1 mm lateral and 0.46 mm posterior to Bregma, and 1.4 mm from the brain surface). Guide cannulae were anchored to the skull by dental cement. After completing surgery stainless steel stylets were inserted into the guide cannula and left in place until injections were made.

### Recording and stimulating procedures

Recording sessions were carried out with three animals at a time. Animals were placed in separate small (5 × 5 × 10 cm^3^) plastic chambers located inside a larger Faraday box (30 × 30 × 20 cm^3^). The electromyographic activity of the orbicularis oculi muscle and the hippocampal field activity were recorded with Grass P511 differential amplifiers (Grass-Telefactor, West Warwick, RI) at a bandwidth of 0.1 Hz–10 kHz. Field EPSP recordings were also made with Grass P511 differential amplifiers through a high-impedance (2 × 1012 Ω, 10 pF) probe. Animals were presented with single square-wave current pulses of different intensities (0.02–0.3 mA). Unless otherwise indicated, single pulses were presented at intensities corresponding to 30–40% of the amount necessary to evoke a saturating response^5^. At the range of intensities used here, no population spikes were observed in the collected recordings. For baseline recordings and to determine the stability of fEPSPs evoked by the electrical stimulation of SCs, single pulses were presented at a rate of 1/20 s. To avoid possible interference with the animal's state of alertness^47^ training sessions lasted for 30 min and were carried out at the same time each day for the same animal. No particular change in the behavioural state of experimental animals was observed across the successive sessions.

### Classical eye-blink conditioning

Classical conditioning was achieved using a trace paradigm (Fig. 2b). A tone (20 ms, 2.4 kHz and 85 dB) was presented as conditioned stimulus, while a 500 μs, 3 × threshold, square and cathodal pulse applied to the supraorbital nerve served as unconditioned stimulus. The shock started 500 ms after the end of the tone. A total of two habituation, 10–12 conditioning, and five extinction sessions were carried out. A conditioning session consisted of 60 tone–shock presentations, and lasted ≈30 min. For a proper analysis of conditioned responses, the tone was presented alone in 10% of the cases. Tone–shock presentations were separated at random by 30±5 s. Animals received one training session per day. For habituation (and extinction) sessions the tone was presented alone 60 times per session, at intervals of 30±5 s. As criteria, we considered a successful conditioned response the presence, during the tone–shock interval, of electromyographic activity lasting >10 ms and initiated >50 ms after tone onset. In addition, the integrated electromyographic activity recorded during the tone–shock interval had to be at least 2.5 times greater than the averaged activity recorded immediately before tone presentation^48^. Field EPSPs were evoked in CA1 during conditioning by presentations of pulses (100 μs, square) applied to SC 300 ms after tone presentation.

### Fear conditioning

Trace fear conditioning experiments took place in chambers (14 × 15 × 12 cm^3^, MED Associates, Georgia, VT) housed in sound-attenuating cubicles. Different visual, olfactory and tactile cues were used to configure different contexts (contexts A, B and C). Animals were placed in individual chambers in a novel context (context A) for 100 s before receiving six tone–foot shock pairings (tone: 20 s, 85 dB, 3 kHz; shock: 2 s, 0.4 mA) with the stimuli separated by a 20-s trace interval. A 100-s inter-trial interval was used. Twenty-four hours after conditioning in context A the animals were placed in the chamber configured as a novel context (context B) and were allowed 100 s to habituate to the context before a 60-s tone (85 dB, 3 kHz) was presented (recall 1). Immediately after recall 1 the animals were removed from the chamber, the plastic floor covering was removed to reveal the shock grid, and the mice were replaced. The mice then received another six tone–foot shock pairings (tone+shock) or only six tones (tone only). A second recall session (recall 2, context C) took place 24 h after recall 1. Freezing, defined as the complete absence of movement except for respiration, was scored manually for each recall session from video recordings and the difference between percentage of time freezing during the tone and percentage of time freezing during the pre-tone period was used as a measure of conditioned response to tone (Δfreezing (%)).

### Viral production

The Venus-P2A-HA-hM4D cassette^25^ was cloned so as to replace the open reading frame of pAAV-Syn-NpHR3.0-EYFP-WPRE (gift of K. Deisseroth, Stanford University, Palo Alto, CA). Production and purification of recombinant AAV (chimeric capsid serotype 1/2) were as described^49^. Viral titres (>10^10^ genomic copies per μl) were determined with QuickTiter AAV Quantitation Kit (Cell Biolabs, San Diego, CA) and RT-PCR^50^.

### Drug administration

The Htr1a selective agonist 8-hydroxy-*N*-[di-*n*-propyl]-aminotetralin (8-OH-DPAT; Sigma-Aldrich, Natick, MA) was dissolved in saline (0.9% NaCl) and injected (subcutaneously in a volume of 5 ml kg^−1^) 15 min before behavioural testing into the scruff of the neck. The non-selective adenosine A1 receptor antagonist, DPCPX (Sigma-Aldrich) was dissolved in 100 mM dimethyl sulfoxide and delivered via bilateral guide cannulae to the dorsal hippocampus (82 nmol per mouse; injection volume: 1.5 μl per hippocampus; flow rate: 0.3 μl min^−1^) 10 min before testing. The selective Npy1r agonist [Pro^30^,Nle^31^,Bpa^32^, Leu^34^]NPY(28–36) was synthesized (ref. 29), dissolved in saline (0.9% NaCl) and delivered via unilateral guide cannula to the right lateral ventricle (20 nmol per mouse; injection volume: 2 μl; flow rate: 1 μl min^−1^) 20 min before testing. The selective hM4D agonist CNO (Enzo Life Sciences, Farmingdale, NY) was dissolved in saline (0.9% NaCl) and systemically injected (3 mg kg^−1^, intraperitoneal) 60 min before testing.

### Histology

At the end of the recording sessions mice were deeply anaesthetized (sodium pentobarbital, 50 mg kg^−1^) and perfused transcardially with saline and 4% phosphate-buffered paraformaldehyde. Brains were dissected, postfixed overnight at 4 °C and cryoprotected in 30% sucrose in PBS. Sections were obtained on a microtome (Leica, Wetzlar, Germany) at 50 μm. Selected sections including the dorsal hippocampus were mounted on gelatinized glass slides and stained using the Nissl technique with 0.1% toluidine blue to determine the location of stimulating and recording electrodes and cannulae bilaterally implanted in dorsal CA1. To determine the location of cannulae implanted into the lateral ventricle mice were infused with 2 μl of 2% cresyl violet dye. Following euthanasia, intact whole brains were rapidly removed from the skull and flash frozen in isopentane. Brain slices were prepared at 30 μm and mounted on glass slides. Correct guide cannula placement was judged by dye staining of the ventricular border. To determine the location of viral infection animals were transcardially perfused (4% paraformaldehyde, 0.1 M phosphate buffer and pH 7.4) and brains removed and left overnight in fixative. Coronal sections (40 μm) were cut on a vibratome (Leica Microsystems). All sections were imaged for Venus and 4,6-diamidino-2-phenylindole fluorescence with a motorized wide-field microscope (Leica Microsystems). No entorhinal cortex-infected animals were excluded from the analysis.

### Data collection and analysis

Electromyographic, extracellular hippocampal activity and 1-volt rectangular pulses corresponding to tone and shock presentations were stored digitally on a computer via an analogue/digital converter (CED 1401 Plus, Cambridge, England) at a sampling frequency of 11–22 kHz and with an amplitude resolution of 12 bits. Data were analysed off-line for quantification of conditioned responses and fEPSP slopes with the help of commercial (Spike 2 and SIGAVG from CED) and custom^5^,^48^ programs. The slope of evoked fEPSPs was computed as the first derivative (V s^−1^) of fEPSP recordings (V). For this, five successive fEPSPs were averaged, and the mean value of the slope during the rise time period (that is, between the initial and final 10% of the fEPSP) was determined. Computed results were processed for statistical analysis using the Sigma Stat software package (SSI, San Jose, CA). Power spectra of hippocampal field activity were computed using fast Fourier transform with a Hanning window expressed as relative power and averaged for each session and/or group. Average power spectra were analysed and compared using the wide-band model, considering theta, beta and gamma bands described previously^51^. Acquired data were analysed using two-way analysis of variance, with days as repeated measure followed by Holm–Sidak *post hoc* testing, or *t*-test.

## Additional information

**How to cite this article:** Madroñal, N. *et al*. Rapid erasure of hippocampal memory following inhibition of dentate gyrus granule cells. *Nat. Commun.* 7:10923 doi: 10.1038/ncomms10923 (2016).

## Supplementary Material

Supplementary Information Supplementary Figures 1-7

## References

[b1] Marr D. Simple memory: a theory for archicortex. Philos. Trans. R. Soc. Lond. B Biol. Sci. 262, 23–81 (1971). 4399412 10.1098/rstb.1971.0078

[b2] Rolls E. T. & Kesner R. P. A computational theory of hippocampal function, and empirical tests of the theory. Prog. Neurobiol. 79, 1–48 (2006). 10.1016/j.pneurobio.2006.04.005 16781044

[b3] Nakashiba T., Young J. Z., McHugh T. J., Buhl D. L. & Tonegawa S. Transgenic inhibition of synaptic transmission reveals role of CA3 output in hippocampal learning. Science 319, 1260–1264 (2008). 18218862 10.1126/science.1151120

[b4] Wang S. H. & Morris R. G. Hippocampal-neocortical interactions in memory formation, consolidation, and reconsolidation. Annu. Rev. Psychol. 61, 49–79 (2010). 19575620 10.1146/annurev.psych.093008.100523

[b5] Gruart A., Muñoz M. D. & Delgado-García J. M. Involvement of the CA3-CA1 synapse in the acquisition of associative learning in behaving mice. J. Neurosci. 26, 1077–1087 (2006). 16436593 10.1523/JNEUROSCI.2834-05.2006 PMC6674570

[b6] Whitlock J. R., Heynen A. J., Shuler M. G. & Bear M. F. Learning induces long-term potentiation in the hippocampus. Science 313, 1093–1097 (2006). 16931756 10.1126/science.1128134

[b7] Davis H. P. & Squire L. R. Protein synthesis and memory. A review. Psychol. Bull. 96, 518–559 (1984). 6096908

[b8] Goelet P., Castellucci V. F., Schacher S. & Kandel E. R. The long and the short of long-term memory–a molecular framework. Nature 322, 419–422 (1986). 2874497 10.1038/322419a0

[b9] Dudai Y. & Morris R. in Brain, Perception, Memory. Advances in Cognitive Sciences ed. Bolhius J. 149–162Oxford University Press (2000).

[b10] Pastalkova E. . Storage of spatial information by the maintenance mechanism of LTP. Science 313, 1141–1144 (2006). 16931766 10.1126/science.1128657

[b11] Levy W. B., Desmond N. L. & Zhang D. X. Perforant path activation modulates the induction of long-term potentiation of the Schaffer collateral–hippocampal CA1 response: theoretical and experimental analyses. Learn. Mem. 4, 510–518 (1998). 10.1101/lm.4.6.510 10701875

[b12] Remondes M. & Schuman E. M. Direct cortical input modulates plasticity and spiking in CA1 pyramidal neurons. Nature 416, 736–740 (2002). 11961555 10.1038/416736a

[b13] Remondes M. & Schuman E. M. Role for a cortical input to hippocampal area CA1 in the consolidation of a long-term memory. Nature 431, 699–703 (2004). 15470431 10.1038/nature02965

[b14] Leutgeb J. K., Leutgeb S., Moser M. B. & Moser E. I. Pattern separation in the dentate gyrus and CA3 of the hippocampus. Science 315, 961–966 (2007). 17303747 10.1126/science.1135801

[b15] Hasselmo M. E. The role of hippocampal regions CA3 and CA1 in matching entorhinal input with retrieval of associations between objects and context: theoretical comment on Lee *et al*. Behav. Neurosci. 119, 342–345 (2005). 15727540 10.1037/0735-7044.119.1.342

[b16] Lassalle J. M., Bataille T. & Halley H. Reible inactivation of the hippocampal mossy fiber synapses in mice impairs spatial learning, but neither consolidation nor memory retrieval, in thevers Morris navigation task. Neurobiol. Learn. Mem. 73, 243–257 (2000). 10775494 10.1006/nlme.1999.3931

[b17] Lee I. & Kesner R. P. Encoding versus retrieval of spatial memory: double dissociation between the dentate gyrus and the perforant path inputs into CA3 in the dorsal hippocampus. Hippocampus 14, 66–76 (2004). 15058484 10.1002/hipo.10167

[b18] Tsetsenis T., Ma X. H., Lo Iacono L., Beck S. G. & Gross C. Suppression of conditioning to ambiguous cues by pharmacogenetic inhibition of the dentate gyrus. Nat. Neurosci. 10, 896–902 (2007). 17558402 10.1038/nn1919 PMC2836794

[b19] Samuels B. A. . 5-HT1A receptors on mature dentate gyrus granule cells are critical for the antidepressant response. Nat. Neurosci. 18, 1606–1616 (2015). 26389840 10.1038/nn.4116 PMC4624493

[b20] Yeckel M. F. & Burger T. W. Feedforward excitation of the hippocampus by efferents from the entorhinal cortex: redefinition of the role of the trisynaptic pathway. Proc. Natl Acad. Sci. USA 87, 5832–5836 (1990). 2377621 10.1073/pnas.87.15.5832 PMC54422

[b21] Berzhanskaya J., Urban N. N. & Barrionuevo G. Electrophysiological and pharmacological characterization of the direct perforant path input to hippocampal area CA3. J. Neurophysiol. 79, 2111–2118 (1998). 9535972 10.1152/jn.1998.79.4.2111

[b22] Delgado-García J. M. & Gruart A. Building new motor responses: eyelid conditioning revisited. Trends Neurosci. 29, 330–338 (2006). 16713636 10.1016/j.tins.2006.05.003

[b23] Izumi Y. & Zorumski C. F. Direct cortical inputs erase long-term potentiation at Schaffer collateral synapses. J. Neurosci. 28, 9557–9563 (2008). 18799687 10.1523/JNEUROSCI.3346-08.2008 PMC2610347

[b24] Armbruster B. N., Li X., Pausch M. H., Herlitze S. & Roth B. L. Evolving the lock to fit the key to create a family of G protein-coupled receptors potently activated by an inert ligand. Proc. Natl Acad. Sci. USA 104, 5163–5168 (2007). 17360345 10.1073/pnas.0700293104 PMC1829280

[b25] Silva B. A. . Independent hypothalamic circuits for social and predator fear. Nat. Neurosci. 12, 1731–1733 (2013). 24212674 10.1038/nn.3573 PMC4194278

[b26] Andersen P., Morris R., Amaral D., Bliss T. & O'Keefe J. The Hippocampus Book Oxford University Press (2007).

[b27] Liang Y. C., Huang C. C. & Hsu K. S. A role of p38 mitogen-activated protein kinase in adenosine A_1_ receptor-mediated synaptic depotentiation in area CA1 of the rat hippocampus. Mol. Brain 1, 13 (2008). 18947392 10.1186/1756-6606-1-13 PMC2579284

[b28] Lein E. S. . Genome-wide atlas of gene expression in the adult mouse brain. Nature 445, 168–176 (2007). 17151600 10.1038/nature05453

[b29] Zwanziger D., Bohme I., Lindner D. & Beck-Sickinger A. G. First selective agonist of the neuropeptide Y_1_-receptor with reduced size. J. Pept. Sci. 12, 856–866 (2009). 19890892 10.1002/psc.1188

[b30] Brun V. H. . Place cells and place recognition maintained by direct entorhinal-hippocampal circuitry. Science 296, 2243–22436 (2002). 12077421 10.1126/science.1071089

[b31] Bhouri M. . mGlu1 receptor-induced LTD of NMDA receptor transmission selectively at Schaffer collateral-CA1 synapses mediates metaplasticity. J. Neurosci. 34, 12223–12229 (2014). 25186764 10.1523/JNEUROSCI.0753-14.2014 PMC4152615

[b32] Kitamura T. . Entorhinal cortical ocean cells encode specific contexts and drive context-specific fear memory. Neuron 87, 1317–1331 (2015). 26402611 10.1016/j.neuron.2015.08.036 PMC5094459

[b33] Aksoy-Aksel A. & Manahan-Vaughan D. The temporoammonic input to the hippocampal CA1 region displays distinctly different synaptic plasticity compared to the Schaffer collateral input *in vivo*: significance for synaptic information processing. Front. Synaptic Neurosci. 23, 5 (2013). 23986697 10.3389/fnsyn.2013.00005 PMC3750210

[b34] Suh J., Rivest A. J., Nakashiba T., Tominaga T. & Tonegawa S. Entorhinal cortex layer III input to the hippocampus is crucial for temporal association memory. Science 334, 1415–1420 (2011). 22052975 10.1126/science.1210125

[b35] Liu X. . Optogenetic stimulation of a hippocampal engram activates fear memory recall. Nature 484, 381–385 (2012). 22441246 10.1038/nature11028 PMC3331914

[b36] Ramirez S. . Creating a false memory in the hippocampus. Science 341, 387–391 (2013). 23888038 10.1126/science.1239073

[b37] Kheirbek M. A. . Differential control of learning and anxiety along the dorsoventral axis of the dentate gyrus. Neuron 77, 955–968 (2013). 23473324 10.1016/j.neuron.2012.12.038 PMC3595120

[b38] Deng W., Mayford M. & Gage F. H. Selection of distinct populations of dentate granule cells in response to inputs as a mechanism for pattern separation in mice. Elife 2, e00312 (2013). 23538967 10.7554/eLife.00312 PMC3602954

[b39] Denny C. A. . Hippocampal memory traces are differentially modulated by experience, time, and adult neurogenesis. Neuron 83, 189–201 (2014). 24991962 10.1016/j.neuron.2014.05.018 PMC4169172

[b40] Sariñana J., Kitamura T., Künzler P., Sultzman L. & Tonegawa S. Differential roles of the dopamine 1-class receptors, D1R and D5R, in hippocampal dependent memory. Proc. Natl Acad. Sci. USA 111, 8245–8250 (2014). 24843151 10.1073/pnas.1407395111 PMC4050601

[b41] Scammell T. E. . Focal deletion of the adenosine A1 receptor in adult mice using an adeno-associated viral vector. J. Neurosci. 23, 5762–5770 (2003). 12843280 10.1523/JNEUROSCI.23-13-05762.2003 PMC6741251

[b42] Pascual O. . Astrocytic purinergic signaling coordinates synaptic networks. Science 310, 113–116 (2005). 16210541 10.1126/science.1116916

[b43] Myers K. M. & Davis M. Mechanisms of fear extinction. Mol. Psychiatry 12, 120–150 (2007). 17160066 10.1038/sj.mp.4001939

[b44] Nader K. & Einarsson E. O. Memory reconsolidation: an update. Ann. N. Y. Acad. Sci. 1191, 27–41 (2010). 20392274 10.1111/j.1749-6632.2010.05443.x

[b45] Paxinos G. & Franklin K. B. J. The Mouse Brain in Stereotaxic Coordinates Academic Press (2001).

[b46] Schwartzkroin P. A. in The Hippocampus eds Isacson R. L., Pribram K. H. 113–136Plenum Press (1986).

[b47] Bramham C. R. & Srebro B. Synaptic plasticity in the hippocampus is modulated by behavioral state. Brain Res. 493, 74–86 (1989). 2776012 10.1016/0006-8993(89)91001-9

[b48] Porras-García E., Cendelin J., Domínguez-del-Toro E., Vožeh F. & Delgado-García J. M. Purkinje cell loss affects differentially the execution, acquisition and prepulse inhibition of skeletal and facial motor responses in Lurcher mice. Eur. J. Neurosci. 21, 979–988 (2005). 15787704 10.1111/j.1460-9568.2005.03940.x

[b49] Pilpel N., Landeck N., Klugmann M., Seeburg P. H. & Schwarz M. K. Rapid, reproducible transduction of select forebrain regions by targeted recombinant virus injection into the neonatal mouse brain. J. Neurosci. Methods 182, 55–63 (2009). 19505498 10.1016/j.jneumeth.2009.05.020

[b50] Knobloch H. S. . Evoked axonal oxytocin release in the central amygdala attenuates fear response. Neuron 73, 553–566 (2012). 22325206 10.1016/j.neuron.2011.11.030

[b51] Múnera A., Gruart A., Muñoz M. D. & Delgado-García J. M. Scopolamine impairs information processing in the hippocampus and performance of a learned eyeblink response in alert cats. Neurosci. Lett. 292, 33–36 (2000). 10996443 10.1016/s0304-3940(00)01430-0

