# Supplementary material for: Rapid erasure of hippocampal memory following inhibition of dentate gyrus granule cells
Source: Nat Commun. 2016 Mar 18;7:10923. doi: 10.1038/ncomms10923 (PMC4802048; doi:10.1038/ncomms10923)
Supplement: Supplementary Information — Supplementary Figures 1-7 [file ncomms10923-s1.pdf]

Supplementary Figure 1

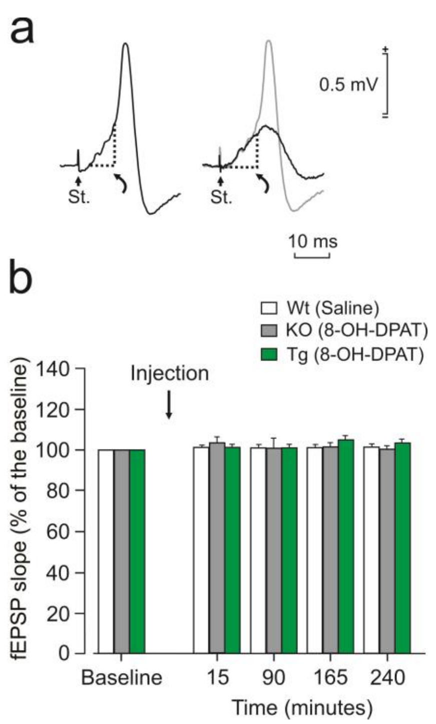

Supplementary Figure 1. Short latency of the fEPSP evoked in CA3 by electrical stimulation of perforant path inputs (**a**) Single and superimposed representative perforant pathway-CA3 fEPSP traces taken before and after 8-OH-DPAT administration in Htr1a^{DG} (Tg) mice (small arrow = stimulation artifact (St.); curved arrow = short latency putative direct PP-CA3 response; see **Fig. 1c-d** for long latency putative disynaptic PP-DG-CA3 response). (**b**) The magnitude of short latency responses was not affected by 8-OH-DPAT administration across wild-type (WT), *Htr1a*^{KO} (KO), or *Htr1a*^{DG} (Tg) mice (mean ± s.e.m.; n = 10; P = 0.477; two-way ANOVA followed by Holm-Sidak *post hoc* test).

Supplementary Figure 2

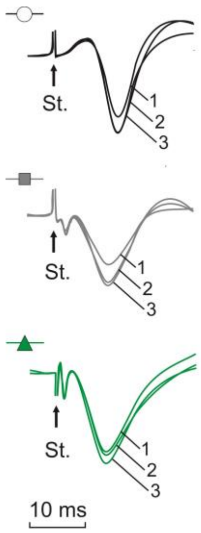

Supplementary Figure 2. Average fEPSC traces during trace eye-blink conditioning. Representative fEPSPs traces recorded in CA1 in response to SC stimulation during habituation (1, day 2), saline or 8-OH-DPAT administration (2, day 8), and recovery (3, day 9) for wild-type (WT, top), *Htr1a*^{KO} (KO, middle) and *Htr1a*^{DG} (Tg, bottom) mice. Numbers are as indicated in Fig. 2e.

Supplementary Figure 3

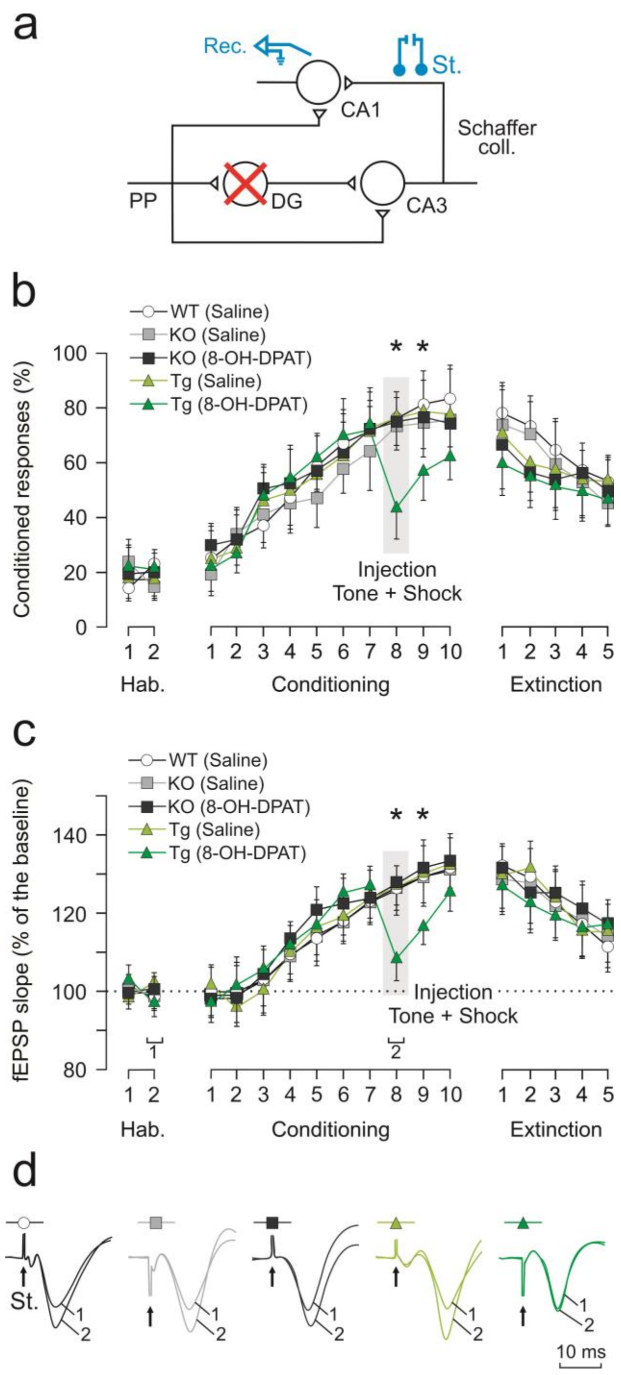

Supplementary Figure 3. No effect of transgene on conditioned behavior or plasticity during eye-blink conditioning. (**a**) As illustrated in Fig. 2a, eye-blink conditioning was followed using EMG measurements of O.O. muscle activity evoked by a tone and by the electrical stimulation of the ipsilateral supraorbitary branch of the trigeminal nerve (Shock). SC plasticity was measured during trace eye-blink conditioning as for Fig. 2 with a recording electrode in CA1 and stimulating electrode in the SC pathway. Mean (**b**) conditioned

responses and (**c**) CA1 fEPSPs, in saline treated wild-type (WT) and vehicle or 8-OH-DPAT (0.3 mg kg^{-1}, s.c.) treated *Htr1a*^{DG} (Tg) and *Htr1a*^{KO} (KO) mice on day 8 of conditioning. Transgenic mice treated with 8-OH-DPAT showed a significant reduction in (**b**) conditioned responding and (**c**) SC plasticity when compared to all other groups that persisted on the day after treatment. (**d**) Representative CA1 fEPSPs corresponding during habituation (1, day 2) and conditioning (2, day 8) for the five groups as indicated in Supplementary Fig. 2c. Trace conditioning was performed as described in Fig. 2 except that conditioning lasted only ten days and was followed immediately by five days of extinction (Tone only presentation). All trials were accompanied by SC stimulation (mean ± s.e.m.; n = 10; *P < 0.05; two-way ANOVA followed by Holm-Sidak *post hoc* test).

Supplementary Figure 4

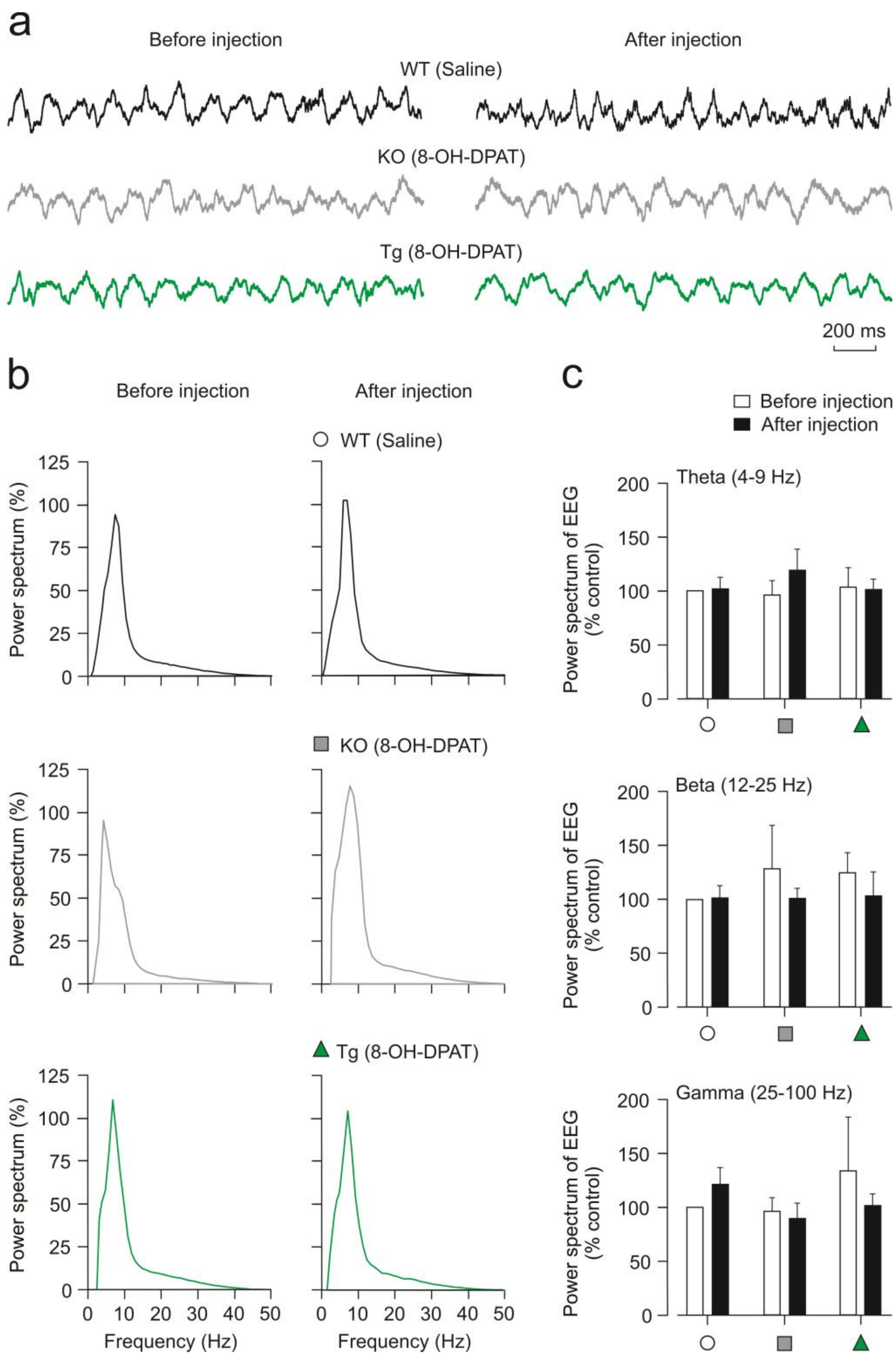

Supplementary Figure 4. CA1 local field potential (LFP) rhythmic activity before and during DG inhibition. (**a**) Representative traces, (**b**) power spectra, and (**c**) mean power in theta (4-9 Hz), beta (12-25 Hz), and gamma (25-100 Hz) frequency domains of CA1 LFP measurements before and after injection of vehicle to wild-type (WT) mice and 8-OH-DPAT

(0.3 mg kg^{-1}, s.c.) to *Htr1a*^{KO} (KO) and *Htr1a*^{DG} (Tg) mice. Prominent spectral power was seen in the theta frequency domain, however no significant differences in spectral power were induced by agonist treatment. Data are derived from mice whose conditioned behavior and plasticity are shown in Supplementary Fig. 2 (mean ± s.e.m.; n = 9-10; two-way ANOVA followed by Holm-Sidak *post hoc* test).

Supplementary Figure 5

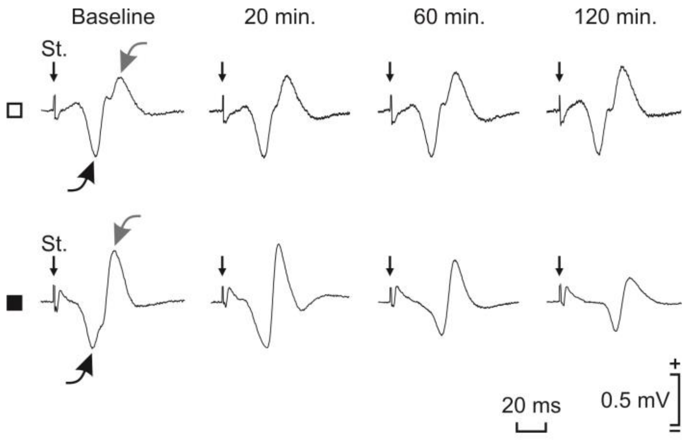

Supplementary Figure 5. fEPSP recorded in CA1 following stimulation of olfactory bulb-entorhinal cortex projections. Representative fEPSPs evoked at CA1 following stimulation of olfactory bulb-entorhinal cortex projections in mice infected with AAV-hM4D and in non-infected (Control) animals before and after systemic administration of CNO. The fast and the slow latency population spike components are indicated (black arrow = short, grey arrow = long).

Supplementary Figure 6

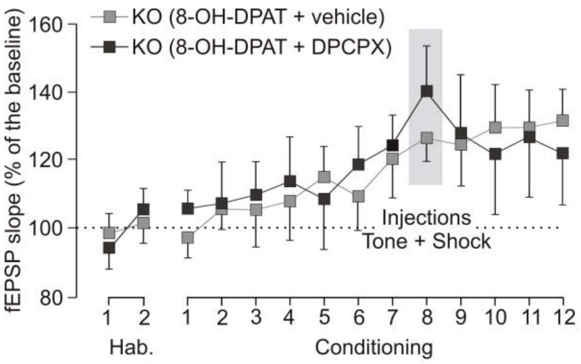

Supplementary Figure 6. **No significant effect of 8-OH-DPAT or DPCPX treatments in *Htr1a*^{KO} mice.** No significant effect of either 8-OH-DPAT (0.3 mg kg^{-1}, s.c.) or the adenosine A1 receptor antagonist DPCPX (bilateral pre-treatment into dorsal CA1; 82 nmol per mouse) on fEPSPs evoked by SC stimulation was seen in *Htr1a*^{KO} (KO) control animals, although there was a trend for DPCPX to increase fEPSPs on the treatment day when compared to vehicle treated mice (day 8; mean ± s.e.m.; n = 7; two-way ANOVA followed by Holm-Sidak *post hoc* test).

Supplementary Figure 7

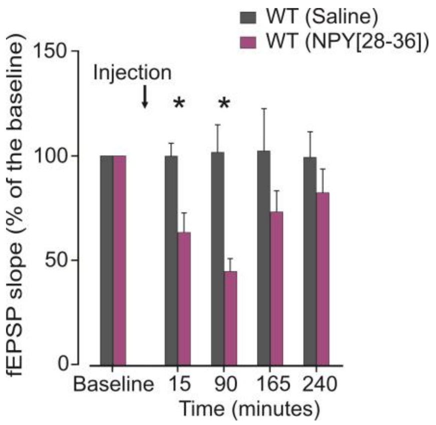

Supplementary Figure 7. Inhibition of DG neurotransmission in wild-type animals. Administration of the Npy1r agonist [Pro^{30}, Nle^{31}, Bpa^{32}, Leu^{34}]NPY(28-36) to wild-type (WT) mice (20 nmoles per mouse, i.c.v.) caused a rapid and selective decrease in long-latency CA3 fEPSPs evoked in anesthetized mice by electrical stimulation of the perforant path when compared to vehicle treated control mice. The suppression of DG neurotransmission reached ~50% and persisted for >2 hours (n = 5-7; *P < 0.05; two-way ANOVA followed by Holm-Sidak *post hoc* test).
